# Supplementary material for: SARS-CoV-2 infection and transmission in the North American deer mouse
Source: Nat Commun. 2021 Jun 14;12:3612. doi: 10.1038/s41467-021-23848-9 (PMC8203675; doi:10.1038/s41467-021-23848-9)
Supplement: Supplementary file 1 — Supplementary Information [file 41467_2021_23848_MOESM1_ESM.pdf]

Supplementary Information

SARS-CoV-2 infection and transmission in the North American deer mouse

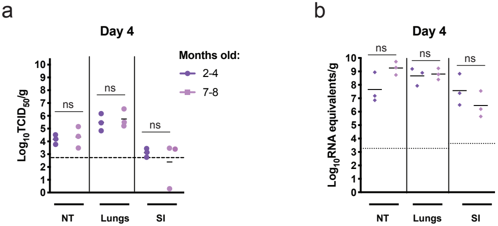

Supplementary Figure 1: Viral burden in the tissues by age. Eight to thirty two-week old male or female deer mice (*P. maniculatus*) were inoculated with $1x10^5$ $TCID_{50}$ of SARS-CoV-2 by an intranasal (i.n.) route of administration. **a**, Infectious viral load in the nasal turbinates, lung, and proximal small intestine at 4 dpi. **b**, Viral RNA load in the nasal turbinates, lung, and proximal small intestine at 4 dpi. Solid lines indicate means. Dashed lines and dotted lines indicate the limit of detection for the TCID50 assay and qRT-PCR assay, respectively. ns = $P > 0.05$, ANOVA with multiple comparison correction.

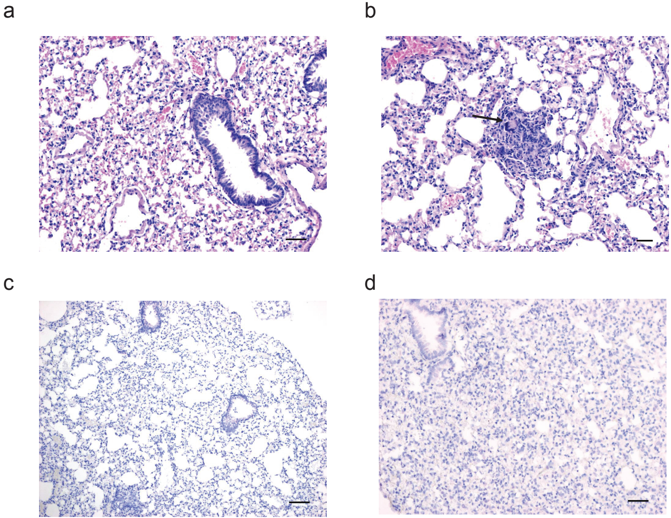

Supplementary Figure 2: Histological analysis of lungs at 21 days following SARS-CoV-2 exposure. Adult male and female deer mice were exposed to 10^5 $TCID_{50}$ SARS-CoV-2 by an i.n. route of infection. Lesions were not observed at this time point (**a**) except for a small focus of inflammation (**b**) with presence of syncytial cell (arrow). ISH using both anti-sense probe (**c**) and sense probe (**d**) was negative. Scale bars = 50 µm (a-b) and 100 µm (c-d). A total of three biological replicates (individual animals) were assessed at each time point, and the selected panels are representative of these findings.

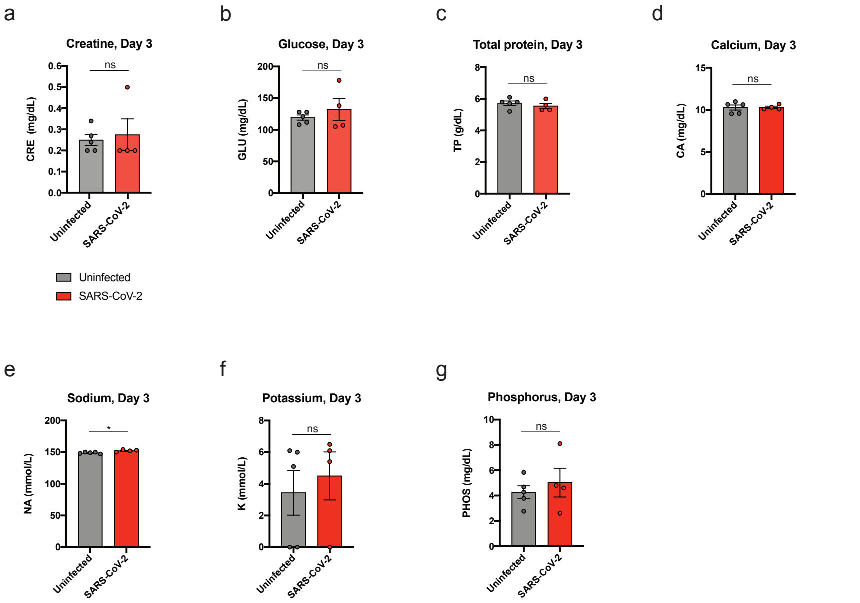

Supplementary Figure 3: Serum biochemical values. Serum biochemistry were measured in uninfected and SARS-CoV-2-infected deer mice at 3 dpi (10^6 $TCID_{50}$, i.n. route). Bars indicate mean, error bars indicate SEM. n = 5/4 biologically independent samples (individual animals) for uninfected/infected. * = $P < 0.05$, ns = $P > 0.05$, two-tailed, unpaired t test with Welch's correction (e, $P = 0.0176$).

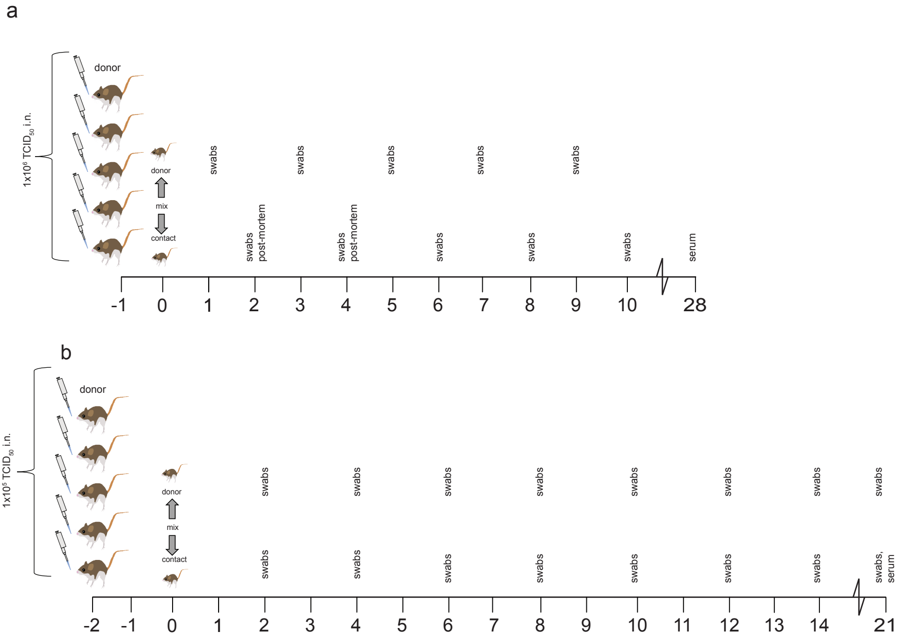

Supplementary Figure 4: Schematic of deer mouse SARS-CoV-2 transmissions study 1 & 2. Adult male and female deer mice were exposed to **a**) $1x10^6$ $TCID_{50}$ or **b**) $1x10^5$ $TCID_{50}$ SARS-CoV-2 by i.n. route of infection. At **a**) 1 dpi or **b**) 2 dpi individual inoculated donor deer mice were transferred to a new cage and co-housed with a single naïve deer mouse (1:1 ratio) to assess SARS-CoV-2 transmission by direct contact. Deer mice were either maintained in direct contact throughout the study for serial swabbing or were humanely euthanized on 2 or 4 dpi for tissue collection.

Figure	Panel	Description	DPI	DM Cohort 1	DM Cohort 2	DM Cohort 3	DM Cohort 4	DM Cohort 5	DM Cohort 6	DM Cohort T1a	DM Cohort T1b	DM Cohort T2
			Dose	$1x10^5$ $TCID_{50}$	$1x10^5$ $TCID_{50}$	$1x10^5$ $TCID_{50}$	$1x10^5$ $TCID_{50}$	N/A	$1x10^6$ $TCID_{50}$	$1x10^6$ $TCID_{50}$	$1x10^6$ $TCID_{50}$	$1x10^5$ $TCID_{50}$
			Uninfected	n = 6	n = 3	N/A	n =3	n = 5	n = 5	N/A	N/A	N/A
			SARS-CoV-2-infected	2-4 months (n =3), 7-8 months (n =3)	n = 6 (12 total)	n = 6	n = 5	N/A	n = 5 (10 total)	infected donor (n = 5), naïve (n = 5)	2x [infected donor (n = 5), naïve (n = 5)]	infected donor (n = 5), naïve (n = 5)
			Sex	M/F	M/F	M/F	M/F	M/F	M/F	M/F	M/F	M/F
Figure	Panel	Description	DPI	0-21/28 dpi	2/4 dpi	0/2/4/6/8 dpi (swabs); 0,7,14,21,28 (blood)	6 dpi	2 dpi	1/3 dpi	-1,1,3,5,7,9 (donor); 4,6 (donor);	2/4 (naïve)	0,2,4,6,8,10,12,14,21
2	a	Survival	0 - 21	X								
2	b	Weight	0 - 21	X								
2	c	Nasal Turbinates	2/4		X							
2	d	Lung	2/4		X							
2	e	Small Intestine	2/4		X							
2	f	Colon	2/4		X							
2	g	Oropharyngeal Swabs	0/2/4/6/8			X						
2	h	Rectal Swabs	0/2/4/6/8			X						
2	i	Nasal Wash*	6				X					
2	j	Urine	6					X		X (donor)		
2	k	Faeces	4					X		X (donor)		
2	l	Blood	1/3					X	X			
2	m	NT/Lung/SI	21	X								
3	a	H&E/ISH NT	2/4		X							
3	b	H&E/ISH Lungs	2/4		X							
4	a	Hematologic Values**	3						X			
4	b	NLR**	3						X			
4	c	ALT	3						X			
4	d	ALB	3						X			
4	e	BUN	3						X			
4	f	Lung Cytokines	2		X			X				
4	g	ELISA	0,7,14,21,28	X (uninfected)		X						
4	h	PRNT90	28	X (uninfected)		X						
5	a	Oropharyngeal Swabs (RNA)	-1 - 10 dpi/dpc							X		
5	b	Rectal Swabs (RNA)	-1 - 10 dpi/dpc							X		
5	c	Oropharyngeal Swabs ($TCID_{50}$)	-1 - 10 dpi/dpc							X		
5	d	Rectal Swabs ($TCID_{50}$)	-1 - 10 dpi/dpc							X		
5	e	Nasal wash, NT, lung	2 dpc								X	
5	f	Nasal wash, NT, lung	4 dpc								X	
5	g	ELISA	21 dpc							X		
5	h	Oropharyngeal Swabs (RNA)	0,2,4,6,8,10,12,14,21									X
5	i	Rectal Swabs (RNA)	0,2,4,6,8,10,12,14,21									X

Supplementary Table 1. Experimental design (Deer mouse cohorts, group sizes, doses).

DM: deer mouse
dpi: days post-infection
NT: nasal turbinates
SI: small intestine
ISH: In situ hybridization
NLR: neutrophil-to-lymphocyte ratio
ALT: alanine aminotransferase
ALB: blood albumin
BUN: blood urea nitrogen
ELISA: enzyme-linked immunosorbent assay
PRNT90: plaque reduction neutralization test 90%
* one sample not obtained
** two samples not obtained

Primer name	Primer/probe sequence
E_Sarbeco_F1	5′- ACAGGTACGTTAATAGTTAATAGCGT-3′
E_Sarbeco_R2	5′-ATATTGCAGCAGTACGCACACA-3′
E_Sarbeco_P1 (probe)	5′-FAM-ACACTAGCCATCCTTACTGCGCTTCG-BBQ-3′

Supplementary Table 2. **Primer/probe sequences**.
FAM: Fluorescein amidite
BBQ: BlackBerry quencher
