# Supplementary material for: SARS-CoV-2 infection and transmission in the North American deer mouse
Source: Nat Commun. 2021 Jun 14;12:3612. doi: 10.1038/s41467-021-23848-9 (PMC8203675; doi:10.1038/s41467-021-23848-9)
Supplement: Supplementary file 3 — Reporting summary [file 41467_2021_23848_MOESM3_ESM.pdf]

nature research

Corresponding author(s): Darwyn Kobasa

Last updated by author(s): Apr 3, 2021

Reporting Summary

Nature Research wishes to improve the reproducibility of the work that we publish. This form provides structure for consistency and transparency in reporting. For further information on Nature Research policies, see our Editorial Policies and the Editorial Policy Checklist.

Statistics

For all statistical analyses, confirm that the following items are present in the figure legend, table legend, main text, or Methods section.

n/a	Confirmed	
☐	☒	The exact sample size (*n*) for each experimental group/condition, given as a discrete number and unit of measurement
☐	☒	A statement on whether measurements were taken from distinct samples or whether the same sample was measured repeatedly
☐	☒	The statistical test(s) used AND whether they are one- or two-sided<br>*Only common tests should be described solely by name; describe more complex techniques in the Methods section.*
☐	☒	A description of all covariates tested
☐	☒	A description of any assumptions or corrections, such as tests of normality and adjustment for multiple comparisons
☐	☒	A full description of the statistical parameters including central tendency (e.g. means) or other basic estimates (e.g. regression coefficient) AND variation (e.g. standard deviation) or associated estimates of uncertainty (e.g. confidence intervals)
☐	☒	For null hypothesis testing, the test statistic (e.g. *F*, *t*, *r*) with confidence intervals, effect sizes, degrees of freedom and *P* value noted<br>*Give P values as exact values whenever suitable.*
☒	☐	For Bayesian analysis, information on the choice of priors and Markov chain Monte Carlo settings
☒	☐	For hierarchical and complex designs, identification of the appropriate level for tests and full reporting of outcomes
☒	☐	Estimates of effect sizes (e.g. Cohen's *d*, Pearson's *r*), indicating how they were calculated

Our web collection on statistics for biologists contains articles on many of the points above.

Software and code

Policy information about availability of computer code

Data collection	Raw data were collected in Microsoft Excel 16.0 (see Source Data file).
Data analysis	Results were analyzed and graphed using Prism 8 software (Graphpad Software). As appropriate, statistical analyses were performed using AN OVA with multiple comparison correction, the multiple t test, or the unpaired t test with Welch's correction or Mann-Whitney test. ACE2 sequences were aligned with Clustal Omega. For the phylogenetic analysis, mitochondrial genome sequences (see Accession codes) were aligned with MAFFT v7.467, with regions of poor alignment trimmed with Gblocks v0.91b resulting in a final alignment of 15,393bp in 115 blocks of minimum 5bp lengths. A maximum likelihood phylogeny was constructed with RAxML v8.2.12 using the GTR+I+G4 substitution model as selected by modeltest-ng,

For manuscripts utilizing custom algorithms or software that are central to the research but not yet described in published literature, software must be made available to editors and reviewers. We strongly encourage code deposition in a community repository (e.g. GitHub). See the Nature Research guidelines for submitting code & software for further information.

Data

Policy information about availability of data

All manuscripts must include a data availability statement. This statement should provide the following information, where applicable:

- Accession codes, unique identifiers, or web links for publicly available datasets
- A list of figures that have associated raw data
- A description of any restrictions on data availability

All of the data supporting the findings of this work can be found within the paper and the accompanying Extended Data files. The raw data are available from the corresponding author upon request or in the corresponding source data file that is provided with this paper.

Field-specific reporting

Please select the one below that is the best fit for your research. If you are not sure, read the appropriate sections before making your selection.

☒ Life sciences ☐ Behavioural & social sciences ☐ Ecological, evolutionary & environmental sciences

For a reference copy of the document with all sections, see nature.com/documents/nr-reporting-summary-flat.pdf

Life sciences study design

All studies must disclose on these points even when the disclosure is negative.

Sample size	Sample sizes were calculated a priori based on estimates of viral burden in tissues and rates of direct contact transmission observed during prior work with related rodent species.
Data exclusions	No animals were excluded from the data analysis although at times sample volumes were insufficient to allow downstream analyses (these instances are outlined in the respective figure legends).
Replication	Data were collected from two independent experiments, and the reported data are representative of these replicates that produced a similar outcome. Experiments are outlined in Extended Data Figure 5.
Randomization	Deer mice were randomly assigned to their respective groups, except for experiments where age was a variable. All downstream samples were therefore randomized for covariates.
Blinding	Blinding of the animal experiments was not performed while experiments were being carried out in containment; however, the resulting samples were assigned numerical sample IDs, and the raw data was not linked to the experimental groups until after the data were generated and were being analyzed.

Reporting for specific materials, systems and methods

We require information from authors about some types of materials, experimental systems and methods used in many studies. Here, indicate whether each material, system or method listed is relevant to your study. If you are not sure if a list item applies to your research, read the appropriate section before selecting a response.

Materials & experimental systems

n/a	Involved in the study	
☐	☒	Antibodies
☐	☒	Eukaryotic cell lines
☒	☐	Palaeontology and archaeology
☐	☒	Animals and other organisms
☒	☐	Human research participants
☒	☐	Clinical data
☒	☐	Dual use research of concern

Methods

n/a	Involved in the study	
☒	☐	ChIP-seq
☒	☐	Flow cytometry
☒	☐	MRI-based neuroimaging

Antibodies

Antibodies used	KPL peroxidase-labeled polyclonal goat antibodies against Peromyscus leucopus IgG (H+L) (Sera Care, cat # 5220-0375).
Validation	"Each lot is tested to assure specificity and lot-to-lot consistency using an in-house ELISA assay.<br>Reference number: 14-33-06" Sera Care website, March 2, 2021.

Eukaryotic cell lines

Policy information about cell lines

Cell line source(s)	VeroE6 (ATCC).
Authentication	The cell line was authenticated by the ATCC, employing morphology, karyotyping, and PCR based approaches to confirm the identity of the cells.
Mycoplasma contamination	Cell lines were confirmed to be negative for Mycoplasma contamination.
Commonly misidentified lines (See ICLAC register)	No commonly misidentified cell lines were used in these studies.

Animals and other organisms

Policy information about studies involving animals; ARRIVE guidelines recommended for reporting animal research

Laboratory animals	Deer mice (Peromyscus maniculatus rufinus) at 8-32 weeks of age were used in these studies. Deer mice were supplied by a breeding colony housed at the University of Manitoba in a pathogen-free facility. All the deer mice were acclimated for a minimum of one week prior to the initiation of experimental procedures. Deer mice were randomly assigned to their respective groups and were housed in a temperature and humidity-controlled, light-cycled facility. Deer mice were supplied with food and water ad libitum and were monitored daily throughout the course of the experiments.
Wild animals	This study did not include animals from the field.
Field-collected samples	N/A
Ethics oversight	The experiments described in this study were carried out at the National Microbiology Laboratory (NML) at the Public Health Agency of Canada. Experiments were approved by the Animal Care Committee located at the Canadian Science Center for Human and Animal Health in accordance with the guidelines provided by the Canadian Council on Animal Care.

Note that full information on the approval of the study protocol must also be provided in the manuscript.
